# Alternative splicing detection workflow needs a careful combination of sample prep and bioinformatics analysis

**DOI:** 10.1186/1471-2105-16-S9-S2

**Published:** 2015-06-01

**Authors:** Matteo Carrara, Josephine Lum, Francesca Cordero, Marco Beccuti, Michael Poidinger, Susanna Donatelli, Raffaele Adolfo Calogero, Francesca Zolezzi

**Affiliations:** 1 Department of Molecular Biotechnology and Health Sciences, University of Torino, Via Nizza 52, 10126 Torino, Italy; 2 Singapore Immunology Network (SIgN), Agency for Science, Technology and Research (A*STAR), Singapore; 3 Department of Computer Science, University of Torino, C.so Svizzera 185, 10149 Torino, Italy; 4 Department of Biological Sciences, National University of Singapore

## Abstract

**Background:**

RNA-Seq provides remarkable power in the area of biomarkers discovery and disease characterization. Two crucial steps that affect RNA-Seq experiment results are Library Sample Preparation (LSP) and Bioinformatics Analysis (BA). This work describes an evaluation of the combined effect of LSP methods and BA tools in the detection of splice variants.

**Results:**

Different LSPs (TruSeq unstranded/stranded, ScriptSeq, NuGEN) allowed the detection of a large common set of splice variants. However, each LSP also detected a small set of unique transcripts that are characterized by a low coverage and/or FPKM. This effect was particularly evident using the low input RNA NuGEN v2 protocol.

A benchmark dataset, in which synthetic reads as well as reads generated from standard (Illumina TruSeq 100) and low input (NuGEN) LSPs were spiked-in was used to evaluate the effect of LSP on the statistical detection of alternative splicing events (AltDE). Statistical detection of AltDE was done using as prototypes for splice variant-quantification Cuffdiff2 and RSEM-EBSeq. As prototype for exon-level analysis DEXSeq was used. Exon-level analysis performed slightly better than splice variant-quantification approaches, although at most only 50% of the spiked-in transcripts was detected. The performances of both splice variant-quantification and exon-level analysis improved when raising the number of input reads.

**Conclusion:**

Data, derived from NuGEN v2, were not the ideal input for AltDE, especially when the exon-level approach was used. We observed that both splice variant-quantification and exon-level analysis performances were strongly dependent on the number of input reads. Moreover, the ribosomal RNA depletion protocol was less sensitive in detecting splicing variants, possibly due to the significant percentage of the reads mapping to non-coding transcripts.

## Background

The application of next-generation sequencing (NGS) to transcriptomics analysis, namely RNA-Seq, has allowed many advances in the characterization and quantification of transcripts. Recently, several developments in RNA-Seq methods have provided an advance in the complete characterization of RNA molecules [1]. These developments included improvements in transcription start site mapping, strand-specific measurements, gene fusion detection, small/long non-coding RNA characterization and detection of alternative splicing events [1]. Further improvements in RNA-Seq methods are allowing transcript quantification from very small amounts of cellular materials or single cells [2-6]. In this work we focused on two of the major steps in RNA-Seq experiments: Library Sample Preparation (LSP) and Bioinformatics Analysis (BA), and their interplay. NGS applications require specific LSP in which fragmented DNA or cDNA molecules are attached to adapters, PCR amplified and sequenced [7]. Since different LSPs can have a significant impact on downstream analysis and interpretation of RNA-Seq results [8], therefore it is evident that robust and unbiased library preparation methods are critical. Nevertheless it has also become clear that LSPs contain biases that compromise the quality of NGS datasets, which can lead to erroneous interpretations [7]. The LSPs available on the market belong to two main classes: i) unstranded and ii) stranded (PolyA*^+ ^*selected, rRNA depleted or low input RNA).

The choice of LSPs does not represent the only critical step in RNA-Seq. Indeed, the sequencing data need to be converted into transcript information (transcript structure, transcript quantification, etc.), and this step requires an accurate selection of the bioinformatics and statistical analysis techniques to be used. The approaches used to quantify known transcripts, i.e. transcripts annotated on the reference genome, and not yet characterized transcripts, i.e. transcripts not associated with an annotation on the reference genome, are different and characterized by different criticalities [9]. In this work we focused only on the annotated splice variants. The BA pipelines for the detection of differentially expressed transcripts are characterized by multiple steps [10], and each of them has an influence on the final results. BA pipelines for differential expression can be divided in two categories: i) differential expression based on splice variant quantification, and ii) exon-based differential expression. This work focused on the interplay of LSP and BA on the statistical detection of AltDE. In detail, we investigated the effect of different LSPs (NuGEN v2, TruSeq unstranded/stranded, ScriptSeq), as well as the effect of PolyA*^+ ^*selection versus ribosomal depletion, on splice variant detection. Furthermore, we compared NuGEN low input protocol with standard TruSeq protocol using BA tools for splice variant-quantification (Cuffdiff [11] and RSEM-EBSeq [12,13]) and for exon-level quantification (DEXSeq [14]).

## Results

We analysed low input RNA (NuGEN) LSP and standard/high input RNA LSP (Illumina TrueSeq and Epicentre ScriptSeq). Sequencing data generated using TrueSeq unstranded PolyA*^+ ^*(100 ngs input total RNA) was used as reference to simplify the comparisons among LSPs. This was because 100 ng of total RNA input material represents the RNA quantity that can be at best obtained from a wide range of biological samples, e.g. animal models, biopsies, FACS sorted cell populations etc. Furthermore, it represents one of the cheaper RNA-Seq experiments available on the market.

### Library sample preparation (LSP) effects on splice variants detection and splice variants characterization

We observed how standard/high (100-1500 ng), low (0.5-2 ng) input protocols, PolyA*^+ ^*selection and ribosomal RNA depletion affect splice variants detection. Specifically, we analysed the LSP effect on splice variants coverage/fragment per kilobase of exon per million reads mapped (FPKM), exons and exon-exon junctions counts. Total RNA, extracted from the mouse dendritic cell line D1 [15], was split in aliquots and converted in libraries using the following sample preparation kits: NuGEN v2, ScriptSeq v1, TruSeq unstranded/stranded (Table 1). The total RNA input material used for NuGEN v2 was 0.5 ng (nu05), 2 ng (nu2) and 100 ng (nu100), while it was 1500 ng for ScriptSeq v1 (ss1500, for short ss), 100 ng (ts100) and 1000 ng (ts1000) for TruSeq unstranded, finally 100 ng for TruSeq stranded (tss100, for short tss). All above-mentioned LSPs were performed after PolyA*^+ ^*selection, but for NuGEN v2 and TruSeq stranded LSP, which was also used in association with the ribo-zero ribosomal RNA depletion (tss_total). For each experimental condition (nu05, nu2, nu100, ss, ts100, ts1000, tss, tss_total) 80 million paired-end reads were collected. The 80 million reads for each condition were assembled combining multiple runs (Additional file 1). We tested the reproducibility among different sequencing runs using deepTools webserver [16]. Correlation between different runs was investigated one chromosome at a time, and the results were reported for chromosome 1 (Additional file 2). Data obtained for the other chromosomes provided similar results (data not shown). Runs clustered on the basis of different LSPs. Ts1000, ts100 and ss cluster together with a Sperman correlation of 0.9. Tss_total clustered together with ts1000, ts100 and ss with a Spearman correlation between 0.77 to 0.83. Tss clustered with ts1000, ts100 and ss with a Spearman correlation between 0.74 to 0.76. NuGEN runs were the least similar, sharing with ts1000, ts100, ss a correlation between 0.60 to 0.67. It was also notable that nu05 runs were very different with respect nu2 and nu100 with a correlation between 0.54 to 0.57.

**Table 1:** Library preparation information

	RNA selection		RNA concentration (ng)				
	
	*Poly A^+^*	*rRNA depletion*	*0.5*	*2*	*100*	*1000*	*1500*
**NuGEN v2**			nu05	nu2	nu100		

**ScriptSeq v1**	✓						ss

**TruSeq unstranded**	✓				ts100	ts1000	

**TruSeq stranded**	✓	✓			tss (PolyA*^+^*) tss_total (rRNA depl)		

**Figure 1:**
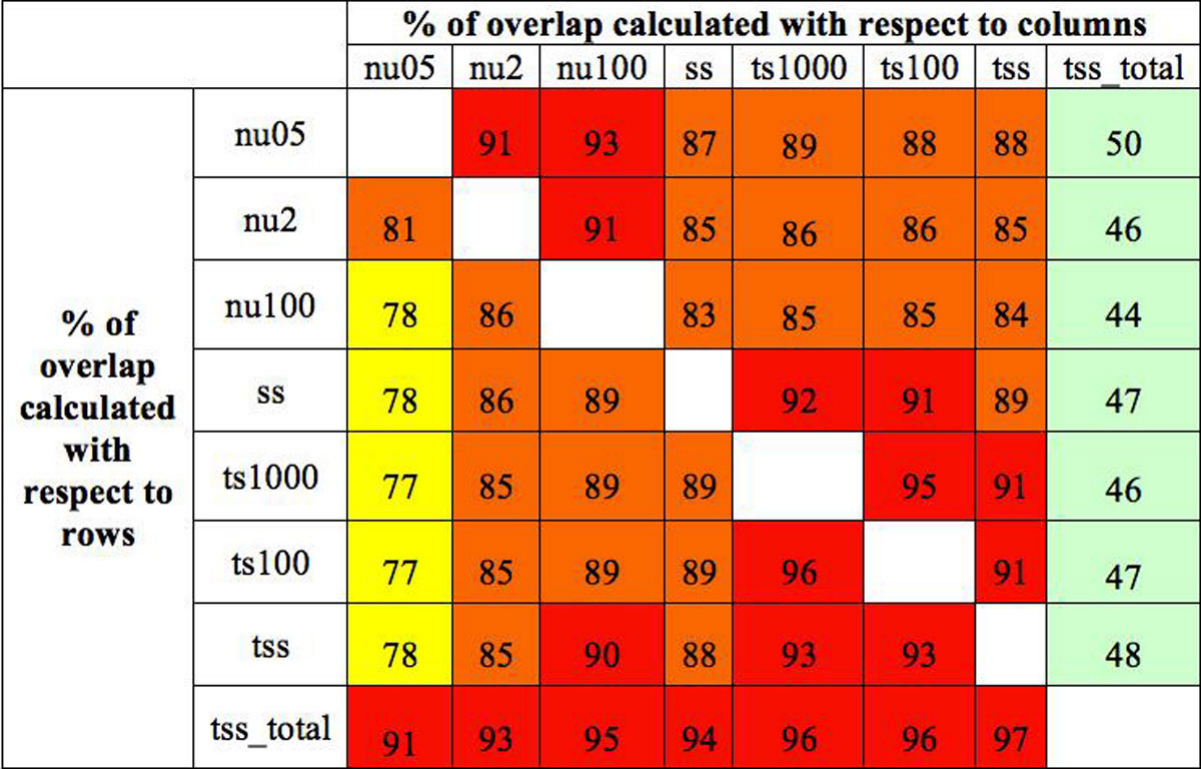
**Percentage of common transcripts (FPKM > 0**.1, Coverage > 0) between different LSPs. Red indicates a percentage greater than 90%. Orange a percentage ranging between 80% and 90%. Yellow a percentage ranging between 70% and 80%. Green is below 70%

Reads were mapped against the mouse genome version 9 (mm9). At least 83% of all reads generated by any of the LSPs could be mapped to the reference genome (Table 2). The percentage of reads characterized by multiple mappings ranged from 7% to 20% in all LSPs but in tss_total, for which it went over 40%. Mapped reads were associated with their corresponding transcript using UCSC annotation and Cufflinks [17], as prototypic method for splice variant quantification. For each experimental condition we retained only the transcripts characterized by FPKM > 0.1 and average coverage > 0 (Table 2). As reported in Figure 1, the number of common detected transcripts was greater than 80% for all LSPs but nu05 and tss_total. The Nu05 shared between 87 to 91% transcripts with the other LSPs but tss_total. Over 90% of tss_total transcripts were detectable by other LSPs (Figure 1). On the other hand less than 50% of the transcripts detected by the other LSPs were also detected by tss_total. Similar observation similarly applied to nu05 even if at a lower extent.

**Table 2:** A) Number of splice variants detected using Cufflinks [17] starting from 80 million reads generated by different Library sample preparation.

Names	KIT	selection	input (ng)	% of single/multiple mapped reads (STAR)	% of mapped reads (STAR)	Splice variants (FPKM > 0.1, Coverage > 0)
nu05	NuGEN	None	0.5	68.49/20.31	88.80	21707

nu2	NuGEN	None	2	80.19/10.07	90.26	24410

nu100	NuGEN	None	100	80.10/9.85	89.95	25901

ss	ScriptSeq	PolyA*^+^*	1500	75.69/7.92	83.61	24135

ts1000	TruSeq unstranded	PolyA*^+^*	1000	87.41/9.20	96.61	24857

ts100	TruSeq unstranded	PolyA*^+^*	100	86.65/9.11	95.76	24701

tss	TruSeq stranded	PolyA*^+^*	100	81.18/14.67	95.85	24318

tss_total	TruSeq Total	Ribo-Zero	100	45.49/41.66	87.15	11993

Ts100 and ts1000, which were the two libraries prepared with TrueSeq unstranded using respectively 100 ng and 1000 ng of input material, allowed the detection of nearly the same number of transcripts (given the chosen minimal threshold of FPKM > 0.1). A possible explanation for the lack of an apparent advantage in using high versus low, PolyA^+ ^RNA input in TruSeq unstranded libraries might be due to transcript quantification sensitivity. Transcripts quantification was done using Cufflinks, because Steijger et al. [18] showed that it provided a good sensitivity with respect to other methods. However, Cufflinks sensitivity was significantly reduced at very low coverage [18]. Thus, it was speculated that, since PolyA^+ ^mRNAs represented a tiny subset of the total input RNA, the increment in input material from 100 ng to 1000 ng was not enough to bring low expressed splice variants in the range of sensitivity of Cufflinks for transcript quantification, given as threshold FPKM > 0.1.

It was notable that the increase of input material affected the overall library yield (Additional file 3). The optimal starting material amount to obtain the higher library yield using TrueSeq (TS) was approx. 200 ng of total RNA. Further increase in the input material significantly reduces the library yield.

**Effect of PolyA^+^ selection versus rRNA depletion**. All LSPs allowed the detection of a similar number of transcripts (Figure 1) except for tss_total, generated using total RNA upon ribosomal depletion. In tss_total, the percentage of transcripts was slightly below 46% even if the total percentage of mapped reads did not differ from the other experiments (Table 2). For this LSP, 41% of the reads were mapped on multiple locations on the genome. This increase in multiple mapping reads was most probably due to non-coding genes, which often are represented in multiple copies in the genome, e.g. tRNAs, miRNA, lincRNA, etc. Coding transcripts undetectable in tss_total were characterized by low coverage/FPKM distributions in ts100, while the transcripts detected both by tss_total and ts100 showed similar coverage and FPKM distributions (Figure 1). We think that, coding transcripts characterized by low expression might not be sampled in tss_total because of the significant reduction of reads mapping to these single copy genes with respect to the other LSPs.

**Transcripts detection in low input protocol**. We observed that the number of detected transcripts in NuGEN v2 depends on the amount of input material (Table 2). It was notable that the library yield increased as the input total RNA increased (Additional file 3). However, since NuGEN is a two-steps protocol, the overall library yield depended also on the amount of cDNA used in the second step (Additional file 3). The number of transcripts in common with the ts100 increased from 0.5 to 100 ng of total RNA input. Moreover, also the number of NuGEN specific transcripts (Table 2 Figure 3A) increased. The coverage of NuGEN detected transcripts (Figure 3B, yellow and green boxes) was lower than ts100 detected transcripts (Figure 3B, violet boxes). This effect was particularly evident for NuGEN specific transcripts (Figure 3B, yellow boxes). However, the behaviour observed for the coverage did not apply to FPKM distribution (Figure 3C). Unless for the nu05 dataset, NuGEN detected transcripts showed FPKM distribution (Figure 3C, yellow/green boxes) similar to that observed for the ts100 dataset (Figure 3C, violet boxes).

**Figure 2:**
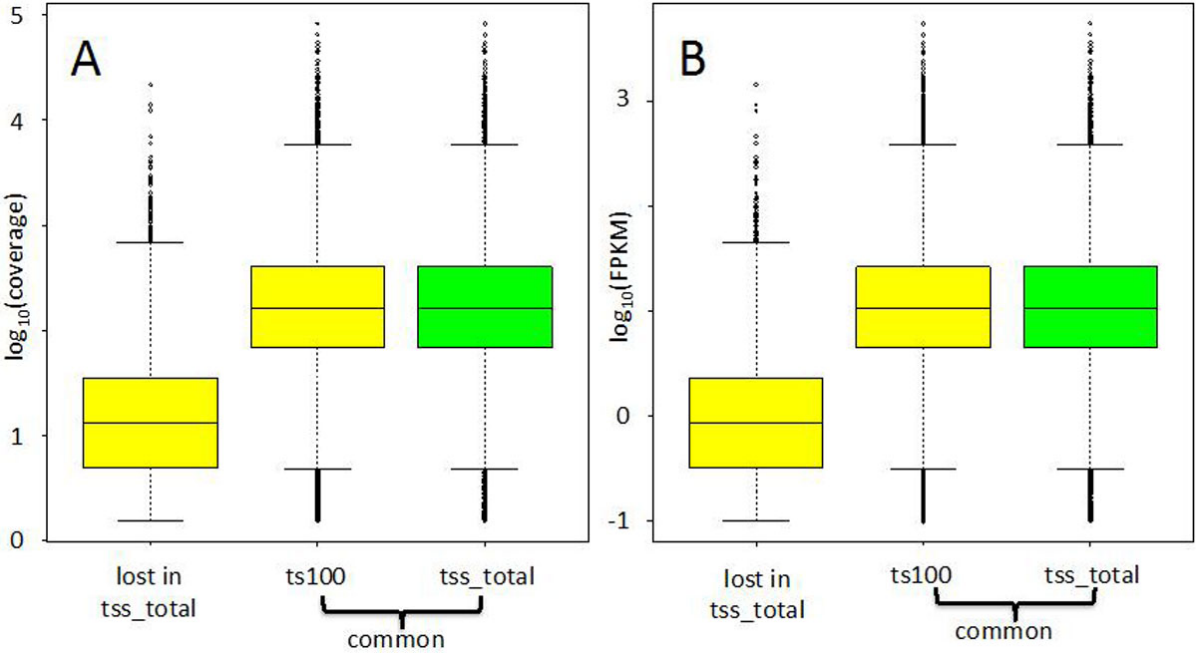
**Coverage and FPKM of splice variants in tss_total**. A) Coverage distributions. The left-most boxplot refers to the coverage observed in ts100 for the set of splice variants that were not detectable in tss_total. The central (tss_total) and the right-most boxplot (ts100) report the coverage distribution for the splice variants that have been detected in both tss_total and ts100. B) As for A, but with respect to FPKM distributions. The left-most boxplot in panel A and B indicate that splice variants undetectable in tss_total are characterized by expression lower than that observable for the subset of the splice variants detectable by both tss_total and ts100. These low expression splice variants were not sampled in tss-total because the number of reads mapping to single copy genes were lower compared to other experiments. FPKM and coverage were calculated at transcript level using Cufflink (2.2.0)

**Figure 3:**
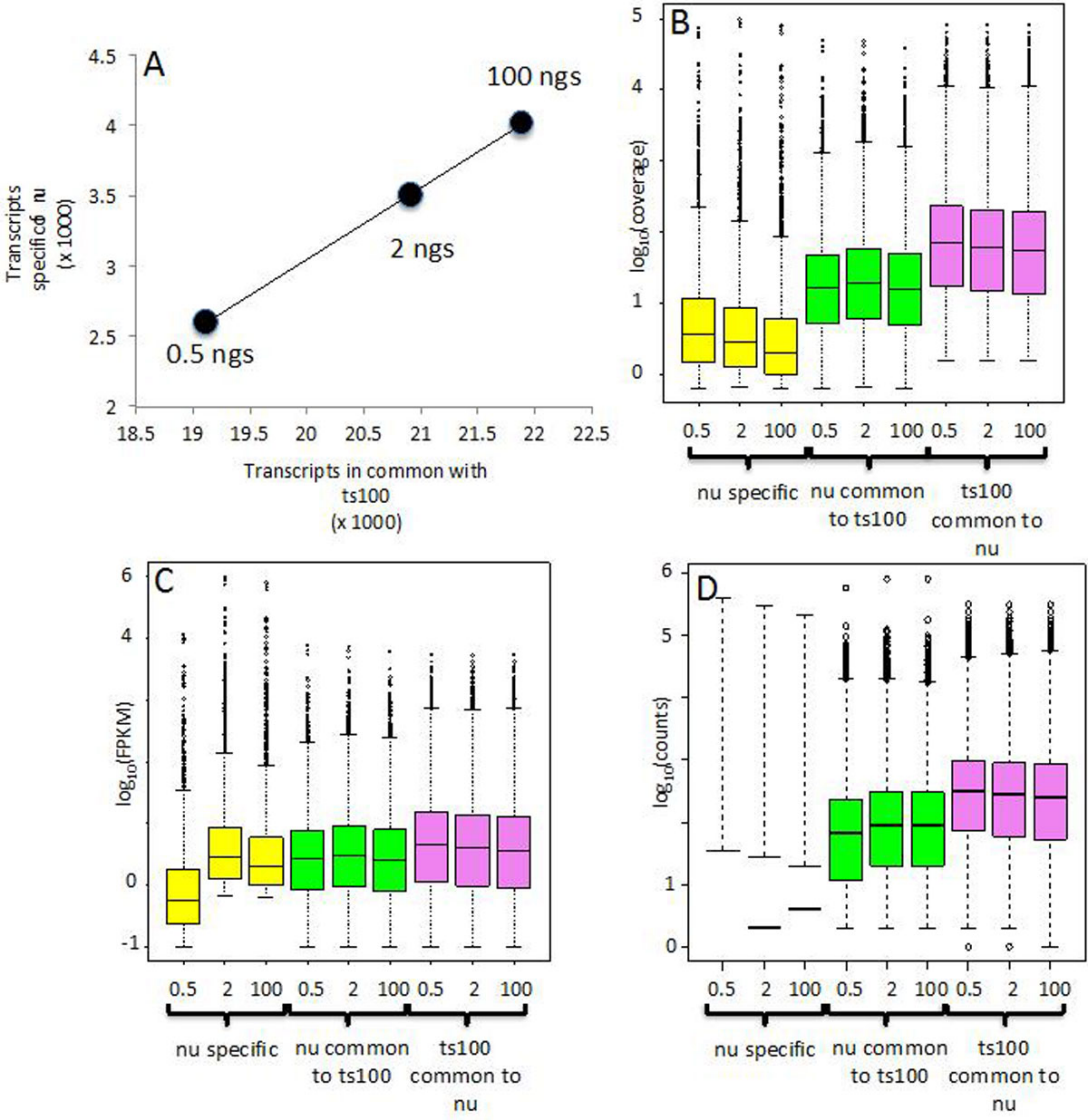
**Transcripts coverage and FPKM in low input LSP**. A) The number of the LSP specific transcripts increases linearly with the increment of the transcripts in common with ts100 LSP, which depends on total RNA input. B) Coverage for transcripts detected only by low input LSP (yellow boxes) is much lower than the coverage of transcripts in common with ts100 (green boxes). The increment on total RNA input does not improve the coverage for transcripts in common with ts100 (green boxes). Coverage in ts100 LSP has higher coverage than the one obtained by low input LSP. C) Unless for 0.5 ng in low input LSP (yellow), the FPKM of all conditions show a similar distribution. D) The counts of the exons associated with the transcripts in B/C indicate a very low exon counts distribution for the nu05, nu2 and nu100 specific-exons (yellow boxes) and a lower number of exon counts associated to transcripts in common with ts100 in nu05, nu2 and nu100 (green boxes) with respect to ts100 exon counts (violet boxes). FPKM and coverage were calculated at transcript level using Cufflink (2.2.0)

**Figure 4:**
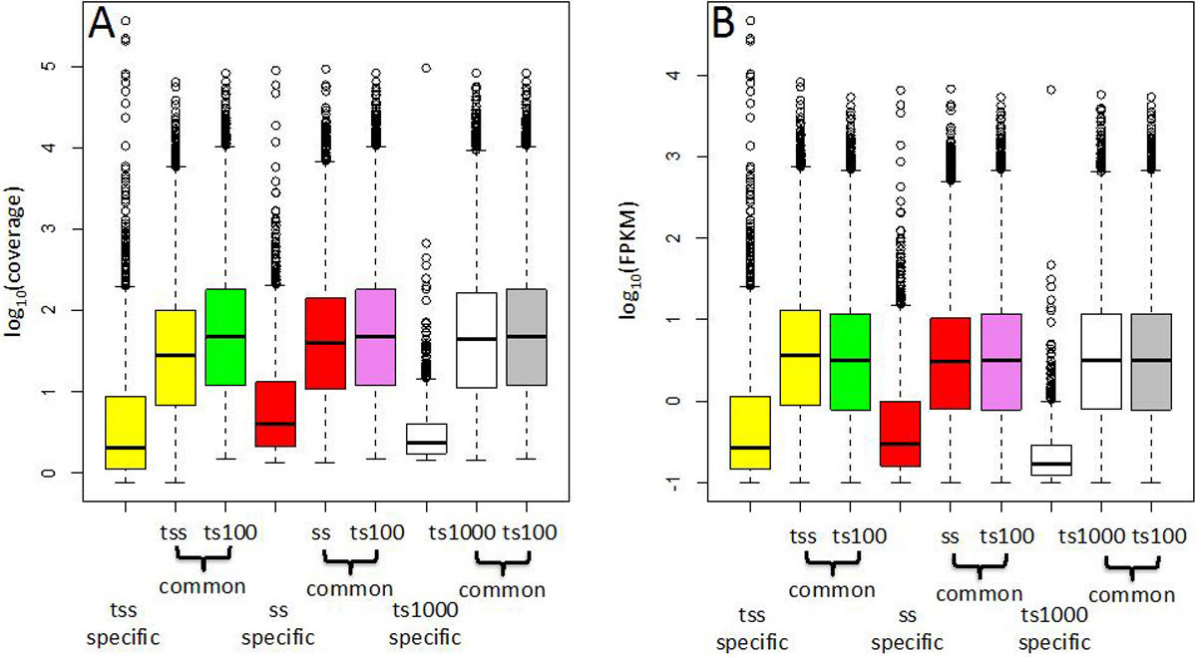
**Transcript coverage and FPKM in LSPs with 100-1000 ng input of total RNA**. A, B, C) Coverage for transcripts detected only by tss, ss, ts1000 LSPs is much lower than the coverage of transcripts in common with ts100. Transcripts detected in common with respect to ts100 show nearly identical coverage. FPKM and coverage were calculated at transcript level using Cufflink (2.2.0)

We analysed coverage and FPKM distributions for ss, tss and ts1000 with respect to ts100 (Figure 4). Coverage and FPKM distributions of transcripts in common between ss, tss, ts1000 and ts100 were very similar to each other. On the other side the LSP specific transcripts were always characterized by very low coverage/FPKM distributions (Figure 4). Thus, the low coverage for LSP transcripts in common with ts100 seemed to be a peculiarity only of data derived from NuGEN LSP.

We further investigated this point by analysing the raw count distribution for exons belonging to the transcripts detected by NuGEN and for those transcripts in common with ts100 (Figure 3D). From this analysis it was clear that exons belonging to transcripts detected by NuGEN, are characterized by low exon coverage (Figure 3D, black boxes). This was particularly evident for the nu05 sample, where the mean of its exon-counts distribution was not shown since the majority of the exons have 0 counts (Figure 3D, black boxes). Instead, a mean value lower than 10 count was observed in samples nu2 and nu100 (Figure 3D, green/violet boxes). For exons detected by both nu and ts100, the exon counts distribution was lower for nu05, nu2, and nu100 (Figure 3D, green boxes) with respect to ts100 (Figure 3D violet boxes). The presence of lower coverage for transcripts/exons detected by NuGEN could represent a critical issue in splice variant differential expression, since it might affect the bioinformatics quantification of the transcripts/exons.

**Figure 5:**
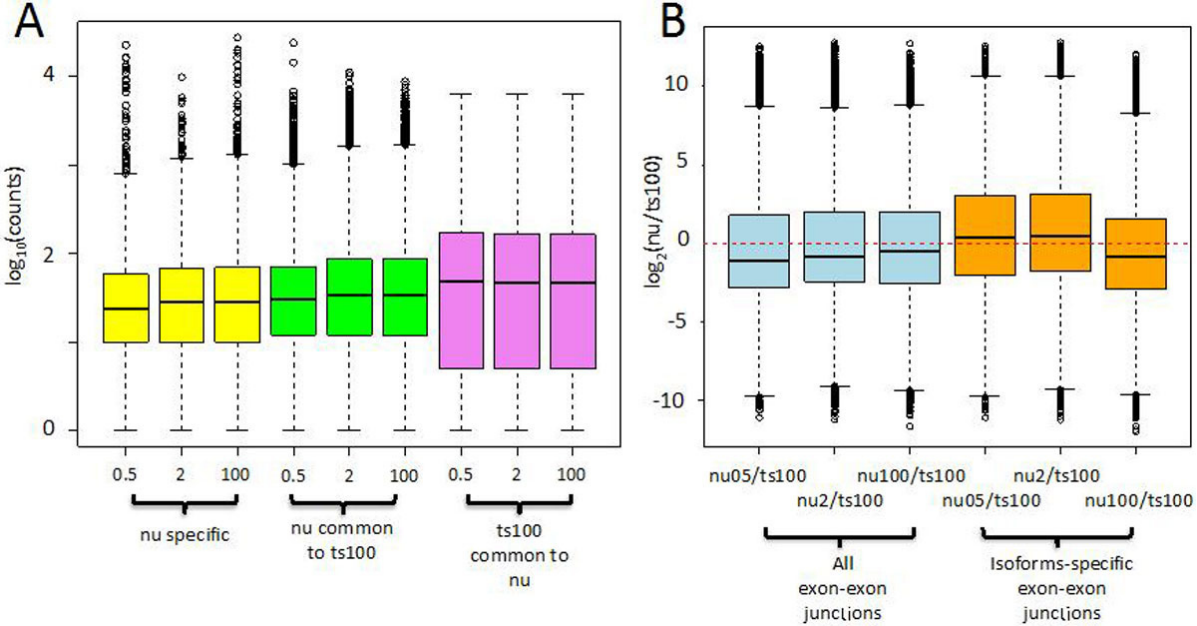
**Characteristics of exon-exon junctions in low input LSP**. A) The number of counts associated with exon-exon junctions in nu05, nu2 and nu 100 both for LSP-specific transcripts and for those transcripts in common with ts100 have detection distributions that differ from those detectable with ts100. B) Log_2 _ratio between nu05, nu2, nu100 and ts100 exon-exon junction counts, for transcripts detected in common by the two LSPs (light blue boxes); log_2 _ratio between nu05, nu2, nu100 and ts100 transcripts-specific exon-exon junction counts (orange boxes). Differences among distributions are not statistically significant.

Finally we checked the presence of detectable differences in the number of exon-exon junctions in transcripts specific for nu05, nu2 and nu100 with respect to those in common with ts100 (Figure 5). The exon-exon junction counts distribution was narrow for transcripts identified using the NuGEN LSP with respect to TruSeq LSP (Figure 5A). The average detection ratio of exon-exon junctions was similar in NuGEN LSP with respect to TruSeq LSP (Figure 5B). Considering only splice variant-specific exon-exon junctions, i.e. exon-exon junctions allowing discrimination between different splice variants, the differences in average detection ratio were also negligible (Figure 5B).

### Benchmark datasets

**Figure 6:**
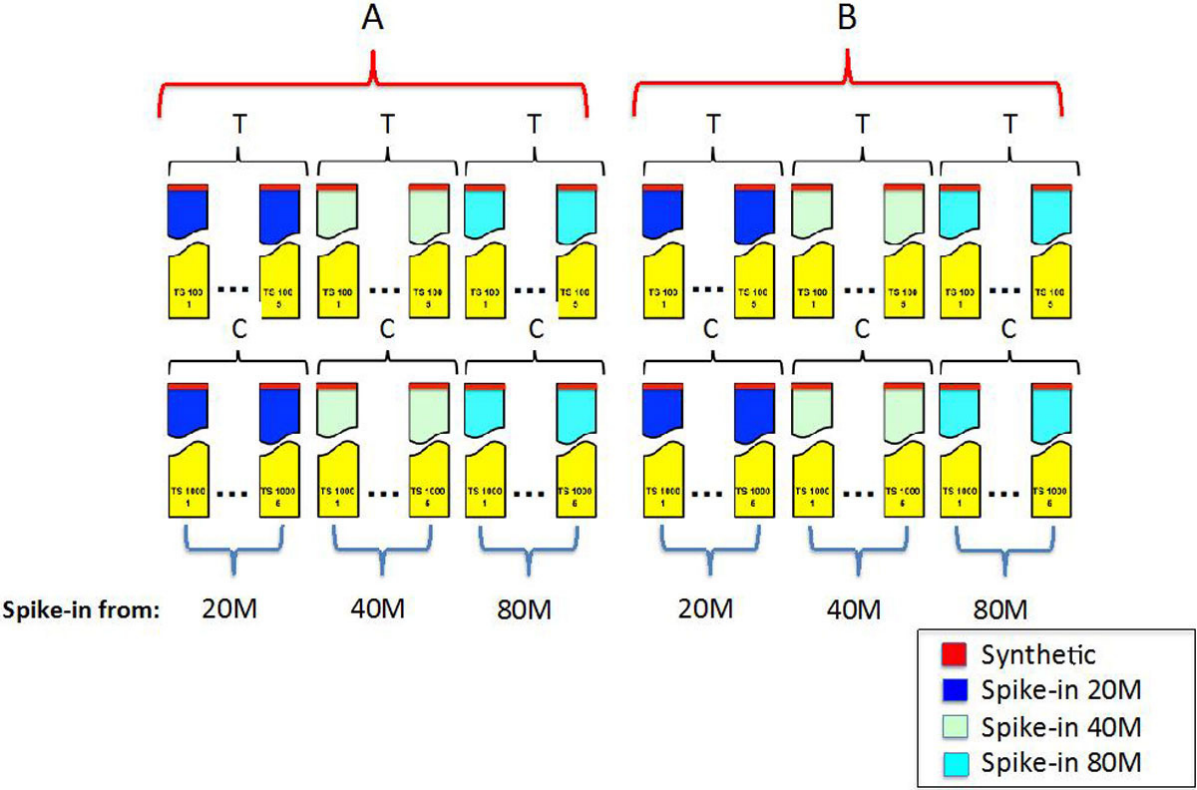
**Benchmark dataset**. A) Three datasets (20TS, 40TS, 80TS), based on spike-in of TruSeq (input: 100 ng total RNA) reads extracted respectively from 20, 40 and 80 million reads were generated using a common background made by 5 different TruSeq library preps having as input 100 ng total RNA (T) and 5 different TruSeq library preps having as input 1000 ng total RNA (C). B) Three datasets (20NU, 40NU, 80NU), based on spike-in of NuGEN (input: 2 ng total RNA) reads extracted respectively from 20, 40 and 80 million reads were generated using the common background described above. Synthetic spikes-in are present both in A and B.

The observations reported in the previous paragraph enlightened that NuGEN v2 influenced transcript quantification in a different way with respect to standard/high input LSPs (TruSeq unstranded/stranded, ScriptSeq). NuGEN protocol using 0.5 ng of input total RNA (nu05) had a very limited ability (-23% with respect to ts100, Figure 1) in detecting splice variants with respect to TruSeq unstranded protocol using 100 ng input total RNA (ts100). The splice variant detection with NuGEN protocol using 2 ng (nu2) or 100 ng (nu100) still remained less efficient than ts100 with respect to the other LSPs. Although nu100 lost, with respect to ts100, only 12% of the detected transcripts (Figure 1) it is not generally used in standard experiments because of the higher complexity/cost of the protocol compared to other LSPs. Nu2 represents the best compromise between the need of a low input RNA quantity and the number of detected splice variants (-16% with respect to ts100, Figure 1). Therefore, we decided to compare the effect of nu2 and ts100 on the detection of differential splice variant expression by BA pipelines. To address this comparison we created benchmark datasets where nu2 and ts100 reads were spiked-in, within a common background made of TruSeq unstranded data (Figure 6, Additional file 4). Specifically, to create the backgrounds C1-C5 and T1-T5 we used reads of 5 technical replicates of ts 100 and 1000 ng of starting material respectively (Additional file 1). The choice of two different backgrounds was driven by the desire of creating a dataset resembling a biologically replicated experiment. However it should be noted that the two backgrounds were extremely similar (Additional file 2 and 7) for the detection of number of exons (Additional file 7A) and expression at exon-level (Additional file 7B).

**Figure 7:**
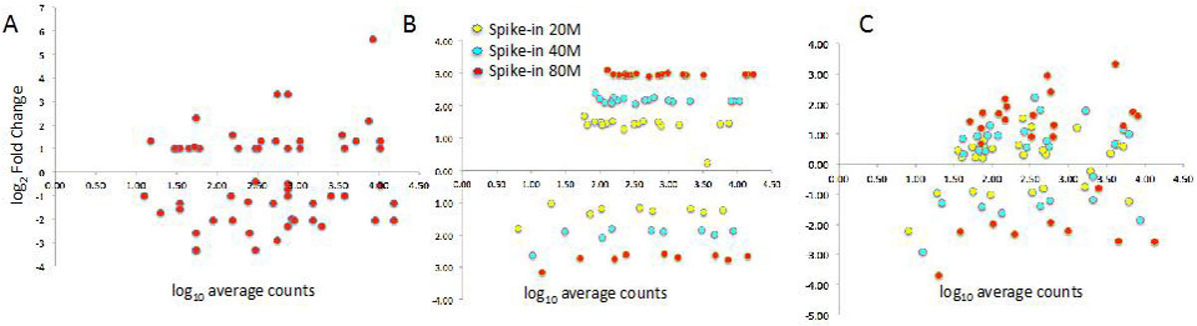
**Differential expression of the spike-in data**. A) Synthetic spikes-in characterized by uniform coverage over the transcripts. B) TruSeq spikes-in. C) NuGEN spikes-in. Y-axes represent average log_2 _fold change between T1-T5 and C1-C5 and X-axes represents average log_10 _counts for the union of T1-T5 and C1-C5. Panel B highlights the homogeneous behaviors of TrueSeq spikes-in with respect to the number of reads used to generate the spikes-in. The differential expression of the spikes-in progressively increases from 20M to 80M reads. The same homogeneous behaviors cannot be detected in NuGEN (panel C)

We spiked-in reads derived from 20, 40 and 80 million reads of both nu2 (20/40/80 NU datasets, Additional file 4) and ts100 dataset (20/40/80 TS, Additional file 5). With this design, we generated a splice variant-level differential expression between C and T groups for 27 transcripts (Figure 7).

Furthermore, to investigate the dependency of the BA approaches on gene-specific splice variants complexity we constructed a synthetic dataset that also included complex expression composition of splice variants for the same gene (Additional file 6). Synthetic reads were characterized by having a uniform distribution over all transcripts and 58 differentially expressed transcripts between C and T groups were generated (Figure 6, 7).

Spikes-in were distributed in both C and T background datasets with respect to two disjoint sets of isoforms, to generate skipping and insertion events resembling biological environmental situation. The differences between backgrounds were negligible (Additional file 2 and 7) therefore we postulated that swapping spikes-in between C and T backgrounds would not affect the alternative splicing detection, as we indeed observed in our experiments (not shown).

All datasets are available at GEO repository with the ID: GSE58001.

### Splice variants differential expression analysis

The identification of differentially expressed splice variants was investigated on the above mentioned datasets using Cuffdiff [11] and RSEM-EBSeq [12,13], as prototypic for direct splice variants-quantification approaches, and DEXSeq [14], as prototypic for exon-level analysis.

The selection of two approaches based on splice variant was motivated by the different normalizations used by Cuffdiff and RSEM-EBSeq. Cuffdiff normalization is based on FPKM [19], while the combination of RSEM and EBSeq allows to use raw counts [13] to estimate differential expression between splice variants.

**Figure 8:**
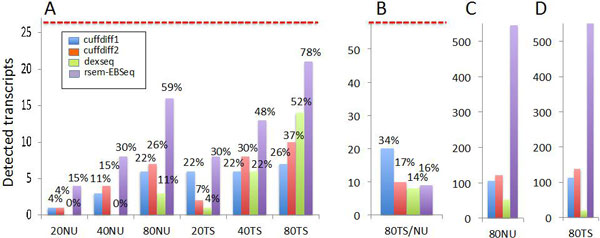
**Statistical detection of spliced variants**. A) True positive transcripts detected as differentially expressed between C1-C5 and T1-T5 groups as function of the spike-ins (20, 40, 80 million reads) and of the LSP (NU, TS) out of 27 spiked-in transcripts. B) True positive synthetic transcripts detected as differentially expressed between C1-C5 and T1-T5 out of the 58 differentially expressed synthetic transcripts with known fold change between T1-T5 and C1-C5. Only the 80 millions spike-in was considered since the synthetic spike-ins are identical over all the datasets. C) False positive transcripts detected in the dataset 80 NU dataset with spike-in derived from 80 million reads, depending on the BA used in the analysis. Only the 80 millions spike-in is considered since the false positive are nearly in the same amount for 20, 40 and 80 millions spikes-in. D) False positive transcripts detected in the TS dataset with spike-in derived from 80 million reads depending on the BA used in the analysis. Only the 80 millions spike-in was considered since the false positive are nearly in the same amount for 20, 40 and 80 millions spikes-in. The numbers on the top of each bar indicates the % of detectable spikes-in.

The increase of the number of reads also increased the detection of differentially expressed splice variants, independently of the dataset under analysis, i.e. 20/40/80NU or 20/40/80TS (Figure 8A). For Cuffdiff we used also the more recent version V2 (Cuffdiff2 for short) that includes the estimation of the over-dispersion due to biological replications [11]. Cuffdiff1 detected a fixed number of transcripts independently of the number of the reads used to generate the spikes-in for the TS dataset (Figure 8A, blue bar). Otherwise, on the NU dataset the differential expression detection increased on the basis of the number of reads used in the spike-in generation. It was notable that, in 80NU dataset, Cuffdiff1 detected the same number of alternative spliced transcripts discovered using 20TS dataset. Thus, the detection in Cuffdiff1 seemed to be quite inefficient when NU datasets were used.

In case of Cuffdiff2, there was an increment in the detected transcripts that was correlated to the number of reads used to generate the spikes-in; this was observable both in TS and NU datasets (Figure 8A, orange bar). Cuffdiff2 detected a greater number of differentially expressed transcripts than Cuffdiff1 in all datasets except for 20TS (Figure 8A, blue and orange bars).

Dillies and Soneson [20,21] have shown that FPKM does not represent the best normalization approach for differential expression analysis. Cuffdiff2 offers two alternative library normalization approaches other than FPKM: geometric and quartile. The geometric normalization is also used in the DESeq. We have repeated the Cuffdiff2 analysis on the 80TS dataset using both geometric and quartile normalization. Although there were some differences in the number of the transcripts detected, the overall number of detected spikes-in was not affected by the different normalization procedures (Additional file 8). The analysis based on RSEM-EBSeq (a tool that works with raw counts) provided a number of detected spikes-in greater than those detected by Cuffdiff2. The difficulties in detecting transcripts differentially expressed in NU datasets were still present also using RSEM-EBSeq but at a lower extent with respect to Cufflink. A similar improvement was not observable for the synthetic transcripts. However, the false positive detection was 6 times greater than Cuffdiff (Figure 8C violet bars).

The exon-level analysis, performed using DEXSeq, was quite inefficient with respect to Cuffdiff (both versions) and REM-EBSeq for the NU dataset (Figure 8A, green bar) and in general in the samples characterized by a low number of input spike-in reads. DEXSeq detected a higher number of differential expressed transcripts compared to Cuffdiff in 80TS (Figure 8A green bar). However, DEXSeq detection rate was lower than RSEM-EBseq (Figure 8A violet bar). Indeed 81% of true spikes-in detected in 80TS by RSEM-EBSeq were associated with a false discovery rate approx. 10 times bigger than the one observed using DEXSeq (Figure 8D).

The experiments performed on the synthetic dataset revealed inferior detection efficiency for all methods (Figure 8B). The best results were obtained by Cuffdiff1, which detected approximately 34% of the total true positive splice variants.

## Discussion

In this work we present the first comparative evaluation of the combined effect of Library Sample Preparation and Bioinformatics Analysis on alternative splicing detection.

Library Sample Preparations using as starting material at least 100 ng of total RNA and undergoing PolyA*^+ ^*enrichment (ss, tss, ts100, ts100), showed a similar behaviour for common detected transcripts. The transcripts detection was significantly impaired for the low expressed transcripts when comparing ribosomal depletion versus PolyA*^+ ^*selection protocols.

Transcripts that were specifically detected only by a single LSP showed poor coverage and they were probably not very informative for splice variant detection, because of the non-uniform coverage at exon-level. As for NuGEN low input protocol, the number of LSP-specific transcripts increased with the rise of the amount of total RNA input. However, those LSP-specific transcripts were characterized by low coverage and in general by very low exon-level counts. FPKM estimation for those transcripts could be misleading, since it had a very similar behaviour to that observed for the transcripts in common with the LSP based on the TruSeq protocol. Nu05, nu2 and nu100 showed a lower coverage with respect to ts100 for the common transcripts as well as for the exon count. The experiments on the benchmark datasets revealed that the lower exon counts generated from NU datasets (20/40/80NU) negatively affected the ability of exon-level based approach (DEXSeq) to detect alternative splicing events. On the other side, when a high number of input reads was used and the preparation was done using the TruSeq protocol (e.g. 80TS dataset), the exon-level based approach provided the best results. Exon-level analysis provided also the best compromise between detection and false discovery rate using the TruSeq protocol.

All our experiments were based on 50-bp paired-end sequences. This choice was a compromise between the performance of the experiment and the sequencing costs. However, the optimal solution, as suggested in the 2011 ENCODE white paper on RNA-seq (https://genome.ucsc.edu/ENCODE/protocols/dataStandards/ENCODE_RNAseq_Standards_V1.0.pdf), would have been 76-bp paired-end sequencing, since the average insert size in the majority of LSP is 150-bp. The use of longer reads could be particularly useful in approaches like TopHat2/Cufflinks, where read splitting is used to detect intron-exon junctions.

## Conclusions

Our results indicate that a low input protocol, such as NuGEN v2, is not suitable for alternative splicing analysis due to the limited coverage at exon-level. Moreover the performances of both splice variants-quantification approaches and exon-level analyses were in general comparable. However, it was notable that, for high number of input spike-in reads, exon-level analysis provided a higher detection rate of alternative splicing events with a reduced level of noise.

Furthermore, we provide to the research community a dataset that can be re-used as benchmark to compare the performance of software devoted to the identification of alternative splicing events. However, because of the intrinsic characteristics of the short sequencing reads, spikes-in generated by extracting reads mapping to a transcript do not necessarily guarantee that they come from that specific transcript. It was impossible to discriminate between different transcripts when reads mapped to exons that were shared between two or more of them. However, since our benchmark dataset contains also synthetic spike-in the combination of the two different types provides a new benchmarking tool to be used to compare software dedicated to alternative splicing detection.

## Materials and methods

### RNA isolation and purification

Total RNA was extracted from D1 mouse cell line [15]. Total RNA was extracted with Trizol Reagent (Invitrogen) followed by RNeasy micro clean-up procedure (Qiagen) as per manufacturer's instructions. Total RNA integrity was assessed by Agilent 2100 Bioanalyzer (Agilent) and the RNA Integrity Number (RIN) was calculated; RNA sample had a RIN = 9.5.

### Library preparation

The following steps were repeated for all library preparations: 1). ERCC RNA Spike in Control Mix 1 (Ambion) was added to D1 total RNA as a first step of each protocol. 2). Two additional rounds of purification of the cDNA libraries were done using Agencourt Ampure XP SPRI beads (Beckman Courter) to remove > 600 bp double stranded cDNA. 3). The length distribution of the cDNA libraries was monitored using DNA 1000 kits on the Agilent Bioanalyzer. 4). Libraries were subjected to an indexed PE sequencing run of 2x51 cycles on an Illumina HiSeq 2000.

#### Illumina TruSeq RNA

Illumina TruSeq RNA Sample Preparation kit version 1 (Low Sample Protocol) was used with slight modifications. Briefly, PolyA*^+ ^*containing RNA molecules were purified using polyT oligo-attached magnetic beads. Thermal fragmentation followed after two rounds of enrichment for PolyA*^+ ^*mRNA. cDNA was synthesized using reverse transcriptase (Superscript II) and random primers. This was followed by second strand cDNA synthesis, end repair process, adenylation of 3' ends and ligation of the adapters. The products were then purified and enriched with 12 cycles of PCR to create the cDNA library.

#### NuGEN Ovation RNA-Seq system version 2 - Dedicated Read Barcode

Total RNA was processed for cDNA synthesis using Ovation RNA-Seq system version 2 (NuGEN Technologies) according to the manufacturer's protocol. Briefly, first strand cDNA was prepared from total RNA using a unique first strand DNA/RNA chimeric primer mix and reverse transcriptase (RT). The primers have a DNA portion that hybridizes either to the 5' portion of the poly(A) sequence or randomly across the transcript. A DNA/RNA heteroduplex double-stranded cDNA was generated by fragmentation of the mRNA within the cDNA/mRNA complex, allowing the DNA polymerase to synthesize a second strand. The DNA then underwent SPIA amplification. SPIA cDNA were sheared to get a size range of 25 bp to 400 bp with the bulk of the material at 150 bp. This was done by sonication (Covaris model S2) with duty cycle 10, intensity 5 and cycle/burst 100 for 300 s. 100 ng and 500 ng of the sheared DNA were used for library preparation using the Encore Rapid Library Systems (NuGEN Technologies) according to manufacturer's protocol; the fragmented DNA underwent end repair, adaptor ligation (with 6 bases indexing tags), and final repair to produce the final library. 4 µl of each purified library underwent 10 cycles of PCR amplification using Illumina TruSeq PCR reagents.

#### Epicentre ScriptSeq v1

PolyA*^+ ^*containing mRNA molecules were selected using RiboMinus™ Eukaryote kit (Life Technologies) for RNA-Seq according to manufacturer's instructions. The RNA samples were chemically fragmented using the StarScript Reverse Transcriptase Buffer and the cDNA Synthesis Primer was annealed to the RNA. 5′ end-tagged cDNA (equivalent to the 3′ end of the original RNA) was produced by random-primed cDNA synthesis. This was followed by 3′**-**Terminal Tagging of the cDNA using the Terminal-Tagging Oligo (TTO) which randomly annealed to the cDNA, including to the 3′ end of the cDNA and served as template for the extension of the cDNA by DNA polymerase. The resulting di-tagged were purified using Qiagen MinElute PCR Purification Kit. Enrichment of the purified di-tagged cDNAs was done with 12 cycles of PCR.

#### Illumina TruSeq Stranded Total RNA

Illumina TruSeq Stranded Total RNA Sample Preparation kit (Low Sample Protocol) was used with slight modifications. The removal of ribosomal RNA was done using Ribo-Zero Gold rRNA removal beads (Epicentre), which deplete samples of both cytoplasmic and mitochondrial ribosomal RNA. After depletion, the RNA was purified and fragmented using divalent cations and thermal fragmentation. First strand cDNA synthesis was performed using reverse transcriptase (Superscript II) and random primers. This was followed by second strand cDNA synthesis using DNA Polymerase I and RNase H and dUTP in place of dTTP. Libraries were prepared as described above for the TruSeq RNA protocol except for the end repair process.

#### Illumina TruSeq Stranded mRNA

Illumina TruSeq Stranded mRNA Sample Preparation kit (Low Sample Protocol) was used with slight modifications. Briefly, polyA containing mRNA molecules were selected using polyT oligo-attached magnetic beads. Fragmentation and library preparation was done as described above for the TruSeq Stranded Total RNA protocol.

### Spikes-in dataset

The common background of the spikes-in dataset was made using paired-end reads generated preparing, with the TruSeq unstranded protocol, 5 libraries, starting with 1000 ng of total RNA extracted from the D1 cell (C1-C5), and 5 libraries starting with 100 ng of total RNA D1 cells (T1-T5) (Additional file 1). The true positive set (TP) of transcripts was defined in the following way: exon counts for samples C1-C5 and T1-T5 were loaded in R using DEXseq package [14] and UCSC mm9 annotation (28232 genes). Genes characterized by at least three splice variants were selected (6582). Then we selected genes having at least one transcript characterized by at least one exon discriminating it from the other splice variants, for a total of 6313 genes. The genes were further filtered, removing all transcripts characterized by having, for the discriminating exons, less than 10 counts in total in C1-C5 and T1-T5 samples (leaving 2970 genes). Out of the 2970 genes 27 were selected, after the inspection of a set of more than a hundred randomly chosen one, and from them one of the splice variant was used for spike-in experiment (Additional file 4). Sequences (generated with NuGEN v2, using as input 2 ng input total RNA, and with TruSeq unstranded, using 100 ng input total RNA), were used to construct three datasets made respectively of 20, 40 and 80 million reads named 20NU, 40NU, 80NU and 20TS, 40TS, and 80TS accordingly. Each dataset was mapped against the mouse genome (mm9) and the reads mapping to the 27 transcripts were extracted and spiked in C1-C5 or in T1-T5 to simulate transcripts up and down-regulation within two experimental conditions (Additional file 4).

It is worth to remark that the selection of the transcripts to be spiked-in is a time-consuming process, due to the complexity in the identification of different types of alternative splicing events (e.g. 5'-end extension, 3'-end reduction, cassette skipping/insertion) for genes having more than one expressed splice variant. Specifically, we selected, as putative targets for spike-in, only those set of splice variant that were expressed both in C and T datasets. This restriction was meant to avoid those cases in which the alternative splicing detection problem reduces to differential gene expression identification.

### Synthetic dataset

Out of the 2970 transcripts 58 were selected, with a similar procedure as above and for each transcript we spiked-in a specific number of reads (Additional file 5). Since spiking-in the identical amount of synthetic reads in all samples of a replication group would represent an oversimplified experimental design, we selected the number of reads to be spiked-in according to a normal distribution, as could be observed in biological replications. We selected the number of reads to be spiked in each sample on the basis of a normal distribution for 10^5 ^elements having a mean equal to the number of reads to be spiked-in, e.g. 100, and a standard deviation 10 times smaller than the mean. For example, we decided to spike-in 100 reads in the 5 replicated of ts100 background (C1-C5), thus 5 random values from a normal distribution of 10^5 ^elements with mean 100 and standard deviation 10 were chosen (e.g. 86, 112, 81, 98, 89) and used to define the number of spike-in reads to be placed in each of the 5 replicates. The required synthetic paired-end reads 2x51nt were constructed to guarantee a uniform distribution both at exon and exon-exon junction level. Reads were then associated with a quality score of 40 and used to generate fastq files. Scripts used to generate the synthetic data set are available upon request.

### Splice variants quantification and statistical detection of alternative spliced variants

Nu05, nu2, nu100, ts100, ts1000, ss, tss, tss_total, C-1-C5 and T1-T5 fastq data were mapped with STAR (2.3.1n) [22] using default settings. For nu05, nu2, nu100, ts100, ts1000, ss, tss and tss_total splice variant quantification was done with Cufflinks (1.3.0 or 2.2.0) [23]. Exon-level quantification was done using DEXSeq (1.10.8) [14] and exon-exon junction quantification was done with subjunc function of the Rsubread (1.14.2) [24] Bioconductor package. Cuffdiff 1 (1.3.0), Cuffdiff2 (2.2.0) [11], RSEM (1.2.15) [12], EBSeq (1.4.0) [13] prototypic BA based on splice variant-quantification, were used for detection of alternative spliced variants between C1-C5 and T1-T5 groups using mm9 UCSC annotation. All Cuffdiff and RSEM-EBSeq analyses were run with standard parameters, unless in the case of the evaluation of the effect of different library normalization procedures done only with Cuffdiff 2 (library-norm-method geometric and library-norm-method quartile). Splice variants were considered differentially expressed if characterized by q-value ≤ 0.05 and FDR ≤ 0.05. for RSEM-EBSeq. Exon-level analysis made use of DEXSeq [14]. Splice variants were considered differentially expressed if at least one splice variant-specific exon was detected as differentially expressed between C1-C5 and T1-T5 groups with a Benjamini & Hochberg adjusted p-value ≤ 0.05.

## Competing interests

None.

## Authors' contributions

CM generated spike-in datasets, LJ made all LSPs, PM performed NGS data QC and mapping, CF executed the bioinformatics comparisons between different LSPs, BM executed the bioinformatics comparisons between different LSP, DS supervised the bioinformatics analysis and revised the paper, CRA investigated the effects of LSPs on BA and wrote the paper, ZF supervised the sample preparations and wrote the paper, CRA and ZF supervised the present work.

## Supplementary Material

Additional file 1 List of datasets generated with different LSPs on the same total RNA sample Click here for file

Additional file 2 reproducibility between different sequencing runs. The datasets used to compare different LSPs were generated combining different sequencing run. Using deepTools webserver [16] we correlated the bam files generated on the same total RNA using different LSPs (Additional file 1). The comparison is reported only on chromosome 1 (the longest one) because of limitation on data uploading per experiment on the deepTools web-server. Click here for file

Additional file 3 **Effect of total RNA input on library yield**. The yield of library is shown with respect to the increment of input total RNA. TrueSeq protocol has a narrow range for the optimal library yield that is about 200 ng (blue dots). The increment of input total RNA for NuGEN protocol resulted in an increment on library yield. The overall yield is dependent also on the amount of cDNA used in the second step of the library preparation (green triangle, red square). Click here for file

Additional file 4 **Background Paired-end reads datasets**. Click here for file

Additional file 7 Exon-level analysis of ts100 and ts1000 dataset used as background for the construction of the spike-in dataset. A) Number of detectable exons, i.e. at least 1 reads mapped of an exon, with respect to the increase of total number of reads. The number of exons detectable by ts100 and ts1000 is very similar, although, over 50 millions reads, ts1000 seems to catch few more exons with respect to ts100. B) Exon-level differential expression calculated comparing the 5 technical replicates from ts100, used as background in T1÷T5, with respect to the 5 technical replicates of ts1000, used as background in C1÷C5. In red are shown the 84 exons detected as differentially expressed between the two groups, FDR ≤ 0.1. In the inset box is shown the distribution of the log_2 _fold change associated to the 84 differentially expressed exons. The two dataset are very similar and even though few exons are detected as differentially expressed their log_2 _fold change difference is negligible. Click here for file

Additional file 5 **Endogenous and spikes-in counts**. Click here for file

Additional file 6 **Synthetic spikes-in data**. Click here for file

Additional file 8 **Effect of different library normalization procedures in Cuffdiff analysis**. Cuffdiff offers the possibility to use three types of library normalization: FPKM, geometic and quartile. Differentially expressed transcripts detected using 80TS dataset. A) 80TS spikes-in. B) Synthetic spikes-in. Click here for file

Additional file 9 Scatter plot of ts100 versus tss. A) log_10_(FPKM). B) log_10_(coverage). Transcripts having in ts100 or tss at least FPKM ≥ 0.1; for level of FPKM lower than 0.1 the value in the plot was set by default to -2. The overall data shows a linear relation both for FPKM and for coverage. Red arrows highlight transcripts that are not correlated in expression in the two LSPs. Click here for file

## Declarations

Publication of this article has been funded by NGS-PTL grant no. 306242.

This article has been published as part of *BMC Bioinformatics *Volume 16 Supplement 9, 2015: Proceedings of the Italian Society of Bioinformatics (BITS): Annual Meeting 2014: Bioinformatics. The full contents of the supplement are available online at http://www.biomedcentral.com/bmcbioinformatics/supplements/16/S9.
