# Supplementary material for: Alternative splicing detection workflow needs a careful combination of sample prep and bioinformatics analysis
Source: BMC Bioinformatics. 2015 Jun 1;16(Suppl 9):S2. doi: 10.1186/1471-2105-16-S9-S2 (PMC4464605; doi:10.1186/1471-2105-16-S9-S2)
Supplement: Additional file 1 — List of datasets generated with different LSPs on the same total RNA sample [file 1471-2105-16-S9-S2-S1.docx]

**Additional file 1:** List of datasets generated with different LSPs on the same total RNA sample

| **Name** | **Library type** | **index** | **Input RNA (ng)** | **HiSeq Lane** |
| --- | --- | --- | --- | --- |
| tss_total_5_1 | tss_total | 5 | 1000 | 1 |
| tss_total_12_1 | tss_total | 12 | 100 | 1 |
| tss_total_6_1 | tss_total | 6 | 100 | 1 |
| tss_total_19_1 | tss_total | 19 | 100 | 1 |
| tss_5_6 | tss | 5 | 100 | 6 |
| tss_6_6 | tss | 6 | 100 | 6 |
| *ts1000_12_2 | ts1000 | 12 | 1000 | 2 |
| *ts1000_12_3 | ts1000 | 12 | 1000 | 3 |
| *ts1000_6_5 | ts1000 | 6 | 1000 | 5 |
| *ts1000_12_5 | ts1000 | 12 | 1000 | 5 |
| *ts1000_6_6 | ts1000 | 6 | 1000 | 6 |
| *ts100_4_2 | ts100 | 4 | 100 | 2 |
| *ts100_6_2 | ts100 | 6 | 100 | 2 |
| *ts100_4_1 | ts100 | 4 | 100 | 1 |
| *ts100_4_3 | ts100 | 4 | 100 | 3 |
| *ts100_4_5 | ts100 | 4 | 100 | 5 |
| ts100_4_6 | ts100 | 4 | 100 | 6 |
| ts100_4_7 | ts100 | 4 | 100 | 7 |
| ss_6_1 | ss | 6 | 1500 | 1 |
| ss_12_1 | ss | 12 | 1500 | 1 |
| ss_6_3 | ss | 6 | 1500 | 3 |
| ss_12_6 | ss | 12 | 1500 | 6 |
| ss_6_7 | ss | 6 | 1500 | 7 |
| ss_12_7 | ss | 12 | 1500 | 7 |
| nu2pcr_4_7 | nu2pcr | 4 | 2 | 7 |
| nu2pcr_3_6 | nu2pcr | 3 | 2 | 6 |
| nu2pcr_1_6 | nu2pcr | 1 | 100 | 6 |
| nu2pcr_2_7 | nu2pcr | 2 | 100 | 7 |
| nu2pcr_5_6 | nu2pcr | 5 | 0.5 | 6 |
| nu2pcr_8_7 | nu2pcr | 8 | 0.5 | 7 |
| nu2pcr_7_6 | nu2pcr | 7 | 0.5 | 6 |
| nu2pcr_6_7 | nu2pcr | 6 | 0.5 | 7 |
| *Libraries used to generate the C and T background respectively. | | | | |
