# Supplementary figures and images for: Alternative splicing detection workflow needs a careful combination of sample prep and bioinformatics analysis
Source: BMC Bioinformatics. 2015 Jun 1;16(Suppl 9):S2. doi: 10.1186/1471-2105-16-S9-S2 (PMC4464605; doi:10.1186/1471-2105-16-S9-S2)

Additional file 3


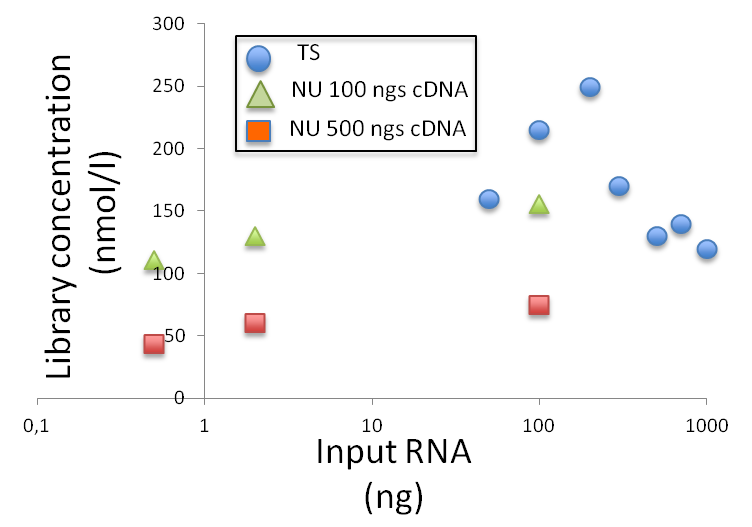

Supplement: Additional file 3 — Effect of total RNA input on library yield. The yield of library is shown with respect to the increment of input total RNA. TrueSeq protocol has a narrow range for the optimal library yield that is about 200 ng (blue dots). The increment of input total RNA for NuGEN protocol resulted in an increment on library yield. The overall yield is dependent also on the amount of cDNA used in the second step of the library preparation (green triangle, red square). [file 1471-2105-16-S9-S2-S3.docx]
