# Supplementary material for: Alternative splicing detection workflow needs a careful combination of sample prep and bioinformatics analysis
Source: BMC Bioinformatics. 2015 Jun 1;16(Suppl 9):S2. doi: 10.1186/1471-2105-16-S9-S2 (PMC4464605; doi:10.1186/1471-2105-16-S9-S2)
Supplement: Additional file 4 — Background Paired-end reads datasets. [file 1471-2105-16-S9-S2-S4.docx]

**Additional file 4:** Background Paired-end reads datasets

|  | **C1** | **C2** | **C3** | **C4** | **C5** | **T1** | **T2** | **T3** | **T4** | **T5** |
| --- | --- | --- | --- | --- | --- | --- | --- | --- | --- | --- |
| **Background PE reads** | 13.4M | 25.8M | 10.9M | 13.4M | 10.0M | 8.4M | 7.4M | 19.6M | 16.1M | 7.3M |
