# Supplementary material for: Alternative splicing detection workflow needs a careful combination of sample prep and bioinformatics analysis
Source: BMC Bioinformatics. 2015 Jun 1;16(Suppl 9):S2. doi: 10.1186/1471-2105-16-S9-S2 (PMC4464605; doi:10.1186/1471-2105-16-S9-S2)
Supplement: Additional file 7 — Exon-level analysis of ts100 and ts1000 dataset used as background for the construction of the spike-in dataset. A) Number of detectable exons, i.e. at least 1 reads mapped of an exon, with respect to the increase of total number of reads. The number of exons detectable by ts100 and ts1000 is very similar, although, over 50 millions reads, ts1000 seems to catch few more exons with respect to ts100. B) Exon-level differential expression calculated comparing the 5 technical replicates from ts100, used as background in T1÷T5, with respect to the 5 technical replicates of ts1000, used as background in C1÷C5. In red are shown the 84 exons detected as differentially expressed between the two groups, FDR ≤ 0.1. In the inset box is shown the distribution of the log2 fold change associated to the 84 differentially expressed exons. The two dataset are very similar and even though few exons are detected as differentially expressed their log2 fold change difference is negligible. [file 1471-2105-16-S9-S2-S7.docx]

Additional file 7
