# Supplementary material for: Alternative splicing detection workflow needs a careful combination of sample prep and bioinformatics analysis
Source: BMC Bioinformatics. 2015 Jun 1;16(Suppl 9):S2. doi: 10.1186/1471-2105-16-S9-S2 (PMC4464605; doi:10.1186/1471-2105-16-S9-S2)
Supplement: Additional file 5 — Endogenous and spikes-in counts. [file 1471-2105-16-S9-S2-S5.docx]

**Additional file 5:** Endogenous and spikes-in counts

|  | **C1-C5 mean** | **nu2** | | | **ts100** | | | **T1-T5 mean** | **nu2** | | | **ts100** | | |
| --- | --- | --- | --- | --- | --- | --- | --- | --- | --- | --- | --- | --- | --- | --- |
| **Splice variant** | **Endogenous** | **20M** | **40M** | **80M** | **20M** | **40M** | **80M** | **Endogenous** | **20M** | **40M** | **80M** | **20M** | **40M** | **80M** |
| **uc009txr.2** | 755 |  |  |  |  |  |  | 607 | 159 | 318 | 541 | 973 | 2047 | 4074 |
| **uc009ghp.2** | 1378.4 |  |  |  |  |  |  | 1068.4 | 1419 | 2637 | 9710 | 1768 | 3641 | 7228 |
| **uc009gho.2** | 2038 | 1368 | 2600 | 10043 | 2623 | 5301 | 10618 | 1566 |  |  |  |  |  |  |
| **uc007jtu.1** | 5453.2 |  |  |  |  |  |  | 4618 | 2781 | 5523 | 10820 | 816 | 16015 | 31665 |
| **uc007jtv.2** | 6860.4 |  |  |  |  |  |  | 5656.6 | 2940 | 5830 | 11437 | 10013 | 19594 | 38859 |
| **uc007gin.1** | 2863.8 | 489 | 974 | 2082 | 4208 | 8428 | 16800 | 2476 |  |  |  |  |  |  |
| **uc008mib.2** | 5186 |  |  |  |  |  |  | 4281.6 | 1263 | 2539 | 6094 | 7315 | 14629 | 29243 |
| **uc009eup.1** | 5863 | 7945 | 15397 | 29280 | 7934 | 15681 | 31240 | 4609.6 |  |  |  |  |  |  |
| **uc007wpv.1** | 659.6 |  |  |  |  |  |  | 521.8 | 202 | 367 | 756 | 951 | 1814 | 3585 |
| **uc007wps.1** | 546.8 | 398 | 722 | 2001 | 752 | 1492 | 3024 | 449.8 |  |  |  |  |  |  |
| **uc012baj.1** | 385 | 346 | 628 | 1088 | 482 | 1000 | 1915 | 280.6 |  |  |  |  |  |  |
| **uc008egx.1** | 358.6 |  |  |  |  |  |  | 261.4 | 362 | 657 | 1135 | 457 | 944 | 1795 |
| **uc012hbj.1** | 299.6 |  |  |  |  |  |  | 234.2 | 135 | 261 | 494 | 396 | 814 | 1578 |
| **uc007dpl.2** | 104.4 | 106 | 219 | 416 | 134 | 261 | 530 | 78 |  |  |  |  |  |  |
| **uc009oda.1** | 117.2 |  |  |  |  |  |  | 93.8 | 15 | 33 | 259 | 166 | 329 | 638 |
| **uc007mki.1** | 142.4 |  |  |  |  |  |  | 109.8 | 49 | 105 | 198 | 206 | 413 | 767 |
| **uc007mkf.1** | 92.6 |  |  |  |  |  |  | 77.4 | 14 | 30 | 47 | 142 | 292 | 540 |
| **uc009fyx.1** | 4.4 | 16 | 29 | 52 | 11 | 23 | 35 | 4 |  |  |  |  |  |  |
| **uc007aui.1** | 100.4 |  |  |  |  |  |  | 75.6 | 55 | 109 | 267 | 134 | 256 | 515 |
| **uc012ffj.1** | 107.6 |  |  |  |  |  |  | 81.4 | 41 | 76 | 180 | 135 | 261 | 546 |
| **uc009gyv.2** | 65.4 | 56 | 108 | 192 | 101 | 212 | 370 | 50.6 |  |  |  |  |  |  |
| **uc007hwe.1** | 70.2 |  |  |  |  |  |  | 64.6 | 31 | 58 | 83 | 118 | 214 | 436 |
| **uc009rfh.2** | 244.4 |  |  |  |  |  |  | 178 | 339 | 653 | 1187 | 255 | 558 | 1150 |
| **uc009rfi.1** | 20.4 | 19 | 29 | 77 | 21 | 55 | 115 | 17.6 |  |  |  |  |  |  |
| **uc009nfj.1** | 399.2 |  |  |  |  |  |  | 306.2 | 75 | 150 | 267 | 582 | 1155 | 2178 |
| **uc009mpg.1** | 61.2 |  |  |  |  |  |  | 51 | 9 | 14 | 117 | 85 | 185 | 350 |
| **uc007ris.1** | 54.2 |  |  |  |  |  |  | 37.8 | 15 | 31 | 63 | 85 | 163 | 289 |
