# Supplementary material for: Alternative splicing detection workflow needs a careful combination of sample prep and bioinformatics analysis
Source: BMC Bioinformatics. 2015 Jun 1;16(Suppl 9):S2. doi: 10.1186/1471-2105-16-S9-S2 (PMC4464605; doi:10.1186/1471-2105-16-S9-S2)
Supplement: Additional file 6 — Synthetic spikes-in data. [file 1471-2105-16-S9-S2-S6.docx]

**Additional file 6:** Synthetic spikes-in data

| **gene** | **splice variant** | **C** | **T** | **avr expr** | **log2FC** |
| --- | --- | --- | --- | --- | --- |
| **kif18a** | **uc008lly.1** | 500 | 1000 | 750 | 1.00 |
| **kif18a** | **uc008llz.1** | 500 | 500 | 500 | 0.00 |
| **kif18a** | **uc008lma.1** | 200 | 200 | 200 | 0.00 |
| **kif18a** | **uc008lmb.1** | 5000 | 5000 | 5000 | 0.00 |
| **kif18a** | **uc008lmc.1** | 5000 | 2500 | 3750 | -1.00 |
| **Dnaja1** | **uc008sht.2** | 100 | 200 | 150 | 1.00 |
| **Dnaja1** | **uc012dby.1** | 100 | 100 | 100 | 0.00 |
| **Dnaja1** | **uc012dbx.1** | 100 | 100 | 100 | 0.00 |
| **Tmco6** | **uc008eog.1** | 1000 | 500 | 750 | -1.00 |
| **Tmco6** | **uc008eoh.1** | 500 | 100 | 300 | -2.32 |
| **Tmco6** | **uc008eoi.1** | 100 | 100 | 100 | 0.00 |
| **Trm1l** | **uc007cys.1** | 5000 | 5000 | 5000 | 0.00 |
| **Trm1l** | **uc007cyw.2** | 500 | 1000 | 750 | 1.00 |
| **Trm1l** | **uc007cyr.1** | 100 | 100 | 100 | 0.00 |
| **Trm1l** | **uc007cyv.2** | 100 | 100 | 100 | 0.00 |
| **Fam126b** | **uc007bcj.2** | 500 | 1000 | 750 | 1.00 |
| **Fam126b** | **uc007bci.2** | 500 | 500 | 500 | 0.00 |
| **Fam126b** | **uc007bch.2** | 100 | 100 | 100 | 0.00 |
| **Mmgt2** | **uc007jjq.1** | 2500 | 1500 | 2000 | -0.74 |
| **Mmgt2** | **uc007jjs.1** | 500 | 100 | 300 | -2.32 |
| **Mmgt2** | **uc007jjr.1** | 100 | 100 | 100 | 0.00 |
| **Prmt10** | **uc009mhp.1** | 100 | 10 | 55 | -3.32 |
| **Prmt10** | **uc012ggg.1** | 100 | 100 | 100 | 0.00 |
| **Prmt10** | **uc009mhq.1** | 100 | 100 | 100 | 0.00 |
| **Thnsl1** | **uc008ind.1** | 100 | 10 | 55 | -3.32 |
| **Thnsl1** | **uc008inb.1** | 100 | 100 | 100 | 0.00 |
| **Thnsl1** | **uc008inc.1** | 100 | 100 | 100 | 0.00 |
| **Tmem8** | **uc009kkr.2** | 10 | 10 | 10 | 0.00 |
| **Tmem8** | **uc009kks.2** | 10 | 10 | 10 | 0.00 |
| **Tmem8** | **uc009kkq.2** | 10 | 50 | 30 | 2.32 |
| **Cbx7** | **uc007wuw.1** | 500 | 1000 | 750 | 1.00 |
| **Cbx7** | **uc007wuv.1** | 100 | 100 | 100 | 0.00 |
| **Cbx7** | **uc007wux.1** | 500 | 500 | 500 | 0.00 |
| **Msh2** | **uc008dvb.1** | 10000 | 7000 | 8500 | -0.51 |
| **Msh2** | **uc008dva.1** | 1000 | 1000 | 1000 | 0.00 |
| **Msh2** | **uc008duz.1** | 100 | 5000 | 2550 | 5.64 |
| **Casp9** | **uc008vpi.1** | 5000 | 2500 | 3750 | -1.00 |
| **Casp9** | **uc012doe.1** | 1500 | 1500 | 1500 | 0.00 |
| **Casp9** | **uc008vph.1** | 10000 | 10000 | 10000 | 0.00 |
| **Rft1** | **uc007svm.1** | 500 | 500 | 500 | 0.00 |
| **Rft1** | **uc007svn.1** | 100 | 100 | 100 | 0.00 |
| **Rft1** | **uc011zhy.1** | 100 | 100 | 100 | 0.00 |
| **Rft1** | **uc011zhz.1** | 1000 | 500 | 750 | -1.00 |
| **Atg16l1** | **uc007bxi.1** | 10 | 100 | 55 | 3.32 |
| **Atg16l1** | **uc007bxk.1** | 300 | 300 | 300 | 0.00 |
| **Atg16l1** | **uc007bxl.1** | 500 | 500 | 500 | 0.00 |
| **Atg16l1** | **uc007bxm.1** | 1000 | 100 | 550 | -3.32 |
| **Atg16l1** | **uc007bxn.1** | 100 | 100 | 100 | 0.00 |
| **Rnf130** | **uc007irk.1** | 100 | 1000 | 550 | 3.32 |
| **Rnf130** | **uc007irl.1** | 3000 | 3000 | 3000 | 0.00 |
| **Rnf130** | **uc007irm.1** | 10 | 10 | 10 | 0.00 |
| **Rnf130** | **uc007irn.1** | 1500 | 200 | 850 | -2.91 |
| **Arpp19** | **uc009qro.1** | 10 | 10 | 10 | 0.00 |
| **Arpp19** | **uc009qrn.2** | 10 | 10 | 10 | 0.00 |
| **Arpp19** | **uc012gwx.1** | 20 | 5 | 12.5 | -2.00 |
| **Arpp19** | **uc009qrp.1** | 5 | 5 | 5 | 0.00 |
| **Tgfbrap1** | **uc007avg.1** | 100 | 100 | 100 | 0.00 |
| **Tgfbrap1** | **uc007ave.1** | 50 | 50 | 50 | 0.00 |
| **Tgfbrap1** | **uc007avh.1** | 20 | 10 | 15 | -1.00 |
| **Tgfbrap1** | **uc007avf.1** | 10 | 10 | 10 | 0.00 |
| **Bcl2l13** | **uc009dns.1** | 20 | 50 | 35 | 1.32 |
| **Bcl2l13** | **uc009dnu.1** | 50 | 20 | 35 | -1.32 |
| **Bcl2l13** | **uc009dnt.1** | 20 | 20 | 20 | 0.00 |
| **Skil** | **uc008ovt.1** | 40 | 80 | 60 | 1.00 |
| **Skil** | **uc008ovu.1** | 30 | 30 | 30 | 0.00 |
| **Skil** | **uc012coi.1** | 10 | 10 | 10 | 0.00 |
| **Skil** | **uc008ovv.1** | 30 | 60 | 45 | 1.00 |
| **tubg1** | **uc007lnl.1** | 20 | 40 | 30 | 1.00 |
| **Slc25a35** | **uc007jop.1** | 200 | 400 | 300 | 1.00 |
| **Ccnt2** | **uc007cky.1** | 4000 | 3000 | 3500 | -0.42 |
| **Gas5** | **uc007det.1** | 10 | 30 | 20 | 1.58 |
| **Hdh** | **uc008xda.1** | 500 | 150 | 325 | -1.74 |
| **Tmem62** | **uc012ccj.1** | 5000 | 10000 | 7500 | 1.00 |
| **Rcbtb2** | **uc007upl.2** | 20 | 90 | 55 | 2.17 |
| **B3gat3** | **uc008gob.1** | 600 | 100 | 350 | -2.58 |
| **Rad51** | **uc012cbp.1** | 6000 | 15000 | 10500 | 1.32 |
| **Bbs9** | **uc012gpo.1** | 600 | 400 | 500 | -0.58 |
| **Sh3pxd2b** | **uc007ijo.1** | 7500 | 3000 | 5250 | -1.32 |
| **Sh3pxd2b** | **uc007ijq.1** | 6000 | 15000 | 10500 | 1.32 |
| **Sh3pxd2b** | **uc007ijp.1** | 6000 | 15000 | 10500 | 1.32 |
| **2810002N01Rik** | **uc007pdt.2** | 6000 | 12000 | 9000 | 1.00 |
| **2810002N01Rik** | **uc011yuy.1** | 25000 | 6000 | 15500 | -2.06 |
| **2810002N01Rik** | **uc007pdu.2** | 25000 | 6000 | 15500 | -2.06 |
| **Pla2g16** | **uc008glj.1** | 750 | 300 | 525 | -1.32 |
| **Pla2g16** | **uc008glh.1** | 600 | 1500 | 1050 | 1.32 |
| **Pla2g16** | **uc008gli.1** | 600 | 1500 | 1050 | 1.32 |
| **AK005305** | **uc007yrp.1** | 600 | 1200 | 900 | 1.00 |
| **AK005305** | **uc007yro.1** | 2500 | 600 | 1550 | -2.06 |
| **AK005305** | **uc007yrn.1** | 2500 | 600 | 1550 | -2.06 |
| **Evi5l** | **uc009kta.1** | 75 | 30 | 52.5 | -1.32 |
| **Evi5l** | **uc009ktc.1** | 120 | 250 | 185 | 1.06 |
| **Dvl3** | **uc007ypv.1** | 60 | 120 | 90 | 1.00 |
| **Dvl3** | **uc007ypx.1** | 250 | 60 | 155 | -2.06 |
| **Dvl3** | **uc007ypw.1** | 250 | 60 | 155 | -2.06 |
| **Sin3b** | **uc012gfz.1** | 120 | 360 | 240 | 1.58 |
| **Sin3b** | **uc009mgq.3** | 120 | 120 | 120 | 0.00 |
| **Sin3b** | **uc009mgp.2** | 2000 | 2000 | 2000 | 0.00 |
| **Sin3b** | **uc009mgo.3** | 120 | 120 | 120 | 0.00 |
| **Phospho2** | **uc008jyr.1** | 360 | 150 | 255 | -1.26 |
| **Phospho2** | **uc008jyp.1** | 150 | 150 | 150 | 0.00 |
| **Phospho2** | **uc008jyq.1** | 500 | 500 | 500 | 0.00 |
| **Zfp384** | **uc009dsw.1** | 1200 | 1200 | 1200 | 0.00 |
| **Zfp384** | **uc009dsx.1** | 100 | 100 | 100 | 0.00 |
| **Zfp384** | **uc009dsy.1** | 1500 | 2500 | 2000 | 0.74 |
| **Zfp384** | **uc009dsz.1** | 2500 | 2500 | 2500 | 0.00 |
| **Tom40** | **uc012fbi.1** | 60 | 10 | 35 | -2.58 |
| **Tom40** | **uc009fna.2** | 10 | 10 | 10 | 0.00 |
| **Tom40** | **uc009fnb.2** | 20 | 20 | 20 | 0.00 |
| **Tom40** | **uc009fnc.2** | 100 | 100 | 100 | 0.00 |
| **Parg** | **uc007syp.1** | 300 | 300 | 300 | 0.00 |
| **Parg** | **uc007syr.1** | 1200 | 400 | 800 | -1.58 |
| **Parg** | **uc007syo.2** | 400 | 400 | 400 | 0.00 |
| **Parg** | **uc007syq.1** | 1200 | 1200 | 1200 | 0.00 |
