# Supplementary material for: Alternative splicing detection workflow needs a careful combination of sample prep and bioinformatics analysis
Source: BMC Bioinformatics. 2015 Jun 1;16(Suppl 9):S2. doi: 10.1186/1471-2105-16-S9-S2 (PMC4464605; doi:10.1186/1471-2105-16-S9-S2)
Supplement: Additional file 8 — Effect of different library normalization procedures in Cuffdiff analysis. Cuffdiff offers the possibility to use three types of library normalization: FPKM, geometic and quartile. Differentially expressed transcripts detected using 80TS dataset. A) 80TS spikes-in. B) Synthetic spikes-in. [file 1471-2105-16-S9-S2-S8.docx]

Additional file 8
