# Supplementary material for: Alternative splicing detection workflow needs a careful combination of sample prep and bioinformatics analysis
Source: BMC Bioinformatics. 2015 Jun 1;16(Suppl 9):S2. doi: 10.1186/1471-2105-16-S9-S2 (PMC4464605; doi:10.1186/1471-2105-16-S9-S2)
Supplement: Additional file 9 — Scatter plot of ts100 versus tss. A) log10(FPKM). B) log10(coverage). Transcripts having in ts100 or tss at least FPKM ≥ 0.1; for level of FPKM lower than 0.1 the value in the plot was set by default to -2. The overall data shows a linear relation both for FPKM and for coverage. Red arrows highlight transcripts that are not correlated in expression in the two LSPs. [file 1471-2105-16-S9-S2-S9.docx]

Additional file 9
